# Invasive Group A Streptococcal Infection in Children, 1992-2023

**DOI:** 10.1001/jamanetworkopen.2025.2861

**Published:** 2025-04-01

**Authors:** Halima Dabaja-Younis, Christopher Kandel, Karen Green, Jennie Johnstone, Zoe Zhong, Caroline Kassee, Vanessa Allen, Irene Armstrong, Mahin Baqi, Kevin Barker, Ari Bitnun, Sergio Borgia, Aaron Campigotto, Sumon Chakrabarti, Wayne L. Gold, Alyssa Golden, Ian Kitai, Julianne Kus, Liane Macdonald, Irene Martin, Matthew Muller, Jeya Nadarajah, Krystyna Ostrowska, Daniel Ricciuto, David Richardson, Medina Saffie, Manal Tadros, Gregory Tyrrell, Monali Varia, Huda Almohri, Shiva Barati, Gloria Crowl, Lubna Farooqi, Maxime Lefebvre, Angel Xinliu Li, Nadia Malik, Mare Pejkovska, Asfia Sultana, Tamara Vikulova, Kazi Hassan, Agron Plevneshi, Allison McGeer

**Affiliations:** 1 Infection Prevention and Control Unit, Sinai Health, Toronto, Ontario, Canada; 2 Department of Laboratory Medicine and Pathobiology, University of Toronto, Toronto, Ontario, Canada; 3 Michael Garron Hospital, Toronto East Health Network, Toronto, Ontario, Canada; 4 Department of Microbiology, Sinai Health, Toronto, Ontario, Canada; 5 Department of Medicine, University of Toronto, Toronto, Ontario, Canada; 6 Toronto Public Health, Toronto, Ontario, Canada; 7 Dalla Lana School of Public Health, Toronto, Ontario, Canada; 8 William Osler Health System, Brampton, Ontario, Canada; 9 Trillium Health Partners, Mississauga, Ontario, Canada; 10 The Hospital for Sick Children, Toronto, Ontario, Canada; 11 Department of Pediatrics, University of Toronto, Toronto, Ontario, Canada; 12 University Health Network, Toronto, Ontario, Canada; 13 National Microbiology Laboratory, Public Health Agency of Canada, Winnipeg, Manitoba, Canada; 14 Public Health Ontario, Toronto, Ontario, Canada; 15 Unity Health, Toronto, Ontario, Canada; 16 Oak Valley Health, Markham, Ontario, Canada; 17 Lakeridge Health, Oshawa, Ontario, Canada; 18 Joseph Brant Hospital, Burlington, Ontario, Canada; 19 Department of Laboratory Medicine and Pathology, Faculty of Medicine and Dentistry, University of Alberta, Edmonton, Alberta, Canada; 20 Region of Peel – Public Health, Brampton, Ontario, Canada; 21 Lifelabs, Toronto, Ontario, Canada; 22 Alberta Precision Laboratories, Edmonton, Alberta, Canada; 23 Li Ka Shing Institute of Virology, University of Alberta, Edmonton, Alberta, Canada

## Abstract

**Importance:**

The resurgence of invasive group A streptococcal (iGAS) infections and progress in GAS vaccine development emphasize the importance of understanding current trends in the epidemiology of iGAS.

**Objective:**

To describe the epidemiology of pediatric iGAS over a 32-year period.

**Design, Setting, and Participants:**

This case series uses population-based surveillance data for iGAS in Toronto and Peel Region, Canada, including *emm* typing from Canada’s National Microbiology Laboratory and population data from Statistics Canada. All children (age <18 years) with iGAS from January 1, 1992, to December 31, 2023, were included. Data were analyzed from July 15, 2023, to September 1, 2024.

**Main Outcomes and Measures:**

Outcomes of interest were disease incidence over time and by age; variation in clinical presentation, disease severity, outcomes and infecting *emm* types; and antimicrobial resistance.

**Results:**

Overall, 498 iGAS cases (300 [60.2%] male; median [IQR] age, 5.1 [2.7-8.6] years) occurred, including 151 (30.7%) in children with comorbidities. The most common presentations were soft tissue infection (140 cases [28.1%]) and bacteremia without focus (131 cases [26.3%]). iGAS incidence increased from 1.8 events per 100 000 population per year in 1992 to 2011 to 2.4 events per 100 000 population per year in 2012 to 2019 (incidence rate ratio, 1.3 [95% CI, 1.1-1.6]), with the increase occurring in GAS infections of the respiratory tract. Incidence declined to 1.2 events per 100 000 population per year in 2020 and 0.5 events per 100 000 population per year in 2021 before increasing to 6.0 events per 100 000 population per year in 2023. In 2022 to 2023, 18 of 56 children with iGAS (32.0%) had a viral respiratory coinfection. Varicella-associated iGAS cases declined from 23 of 137 children (16.8%) in 1992 to 2001 to 2 of 223 children (0.9%) in 2012 to 2023 (*P* < .001), after routine varicella vaccination implementation in 2004. Streptococcal toxic shock syndrome occurred in 29 children (5.8%), necrotizing fasciitis in 12 children (2.4%); 10 children (2.0%) died. The most common *emm* types were *emm*1 (182 of 471 isolates [38.6%]), *emm*12 (75 isolates [15.9%]*),* and *emm*4 (31 isolates [6.6%]). The M1_UK_ subtype was first identified in 2019 and comprised 32 of 46 *emm*1 isolates (70.0%) from 2019 to 2023. Compared with other *emm* types, *emm*1 was more likely to be associated with pneumonia (odds ratio [OR], 1.99 [95% CI, 1.16-3.40]), bone and joint infections (OR, 1.70 [95% CI, 1.08-2.68]), and intensive care unit admission (OR, 1.67 [95% CI, 1.03-2.68]); *emm*4 was more likely to be associated with bacteremia without focus (OR, 6.10 [95% CI, 2.83-13.16]). Overall, 437 isolates (92.8%) were of *emm* types included in the 30-valent GAS vaccine.

**Conclusions and Relevance:**

This case series found that pediatric iGAS incidence increased in south-central Ontario prior to and after the COVID-19 pandemic in association with increased iGAS infections of the respiratory tract. Respiratory viral coinfections were common. Different *emm* types were associated with differing presentations and severity.

## Key Points

**Question:**

How has the epidemiology of invasive group A streptococcal (iGAS) infection in children in Ontario, Canada, changed since 1992?

**Findings:**

This case series from population-based surveillance of 498 pediatric iGAS infections in Ontario, Canada, found that iGAS incidence increased after 2010 in association with an increase in iGAS pneumonia. Viral coinfections were common, different *emm* types varied in incidence over time and were associated with different clinical presentations and severity of illness, and more than 90% of isolate types were included in a 30-valent GAS vaccine currently in development.

**Meaning:**

These findings suggest that iGAS infections are increasing in Ontario and that respiratory viral and GAS vaccines may be protective.

## Teaser

The case series describes the epidemiology of pediatric invasive group A streptococcal infections in Toronto and Peel region of Canada from 1992 to 2023.

## Introduction

Group A *Streptococcus* (*Streptococcus pyogenes*; GAS) is a gram-positive pathogen associated with high morbidity and mortality. Its most severe acute manifestation is invasive GAS (iGAS) infection, which can be associated with streptococcal toxic shock syndrome (STSS), necrotizing fasciitis (NF), and death.^1^ The clinical presentations of GAS disease have varied over time and across regions. In the late 1980s, Stevens et al^2^ first reported increased severity of iGAS infections and scarlet fever in North America, associated with *emm*1 infections. After a period of apparent stability, several jurisdictions reported further increases in iGAS incidence between 2014 and 2019.^3,4,5,6^ During the COVID-19 pandemic, iGAS incidence declined; however, many jurisdictions have reported a postpandemic surge, particularly in children.^7,8,9,10,11^ Over time, changes in incidence and severity have often been attributed to the emergence of new more virulent strains of *S pyogenes*.^6,8,9^

Since 1992, the Toronto Invasive Bacterial Diseases Network (TIBDN) has performed population-based surveillance for iGAS in Toronto and Peel Region in south-central Ontario, Canada. We evaluated changes in the incidence, clinical features, and microbiologic characteristics of iGAS among children over a 32-year period.

## Methods

This case series was approved with waiver of consent by the research ethics boards of all participating institutions in accordance with established ethical guidelines for human research involving medical record reviews. This study is reported following the Strengthening the Reporting of Observational Studies in Epidemiology (STROBE) reporting guideline.

### Population-Based Surveillance

From 1992 to 2023, TIBDN performed population-based surveillance for iGAS in Toronto and Peel Region, an urban/suburban area with a population of 4.5 million in 2020. Residence in the population area was defined by postal code. The laboratory-based surveillance involved all 28 hospitals providing care to and all 25 laboratories processing sterile site cultures from residents of the population area. Laboratory personnel notified the study office whenever GAS was isolated from sterile site specimens. Laboratories were audited annually to ensure complete reporting. This analysis included only pediatric cases (age <18 years).

### Laboratory Procedures

Participating laboratories sent isolates to the TIBDN laboratory at Mount Sinai Hospital for confirmation of *S pyogenes*. M/*emm* typing was performed at the National Centre for Streptococcus in Edmonton, Alberta, or the National Microbiology Laboratory in Winnipeg, Manitoba. Serologic M typing was transitioned to sequencing of the M-specific region of the *emm* gene in 2006, then to whole genome sequencing (WGS) in 2022. WGS used the Illumina platform and identified *emm* type via the WGS Analysis and Detection of Molecular Markers pipeline and the M1_UK_ lineage, as previously described.^6,12,13,14^ Antimicrobial susceptibility testing was performed either by disc diffusion following Clinical Laboratory Standards Institute guidelines, or by prediction from WGS.^14,15^

### Data Collection and Definitions

Clinical and demographic data, including results of concurrent viral respiratory testing, were obtained by medical record review. iGAS was defined using the Canadian case definition: systemic illness occurring in association with the isolation of GAS from a normally sterile body fluid or site (eg, blood, synovial fluid, surgical tissue or swabs) or from nonsterile specimens if associated with STSS or NF.^16^ Primary site of infection was based on attending physician documentation.

Underlying comorbidities were as documented in medical records. Immunosuppression included both immunocompromising conditions and chronic receipt of immunosuppressive therapy, as previously described.^17^ STSS was defined as hypotension and at least 2 of acute kidney failure, coagulation or liver abnormalities, acute respiratory distress syndrome, rash, or soft tissue necrosis.^16^ Death associated with iGAS was defined as that occurring during hospitalization and within 30 days of iGAS onset.^18^

### Statistical Analysis

Data were entered and cleaned using SAS software version 9.4 (SAS Institute) or REDCap (Vanderbilt University) and analyzed using SPSS software version 26 (IBM). Proportions were compared using χ^2^ or Fisher exact tests, with exact 95% CIs for odds ratios (ORs). The incidence of iGAS by age group was calculated using age-specific population estimates from Statistics Canada.^19^ Incidence over time was compared using incidence rate ratios (IRRs) with 95% CIs.^20^ Incidence by *emm* type was adjusted for cases with missing isolates by assuming that the *emm* type distribution in missing isolates was the same as for that in typed isolates in each year. *P* values were 2-sided, and statistical significance was set at *P* < .05. Data were analyzed from July 15, 2023, to September 1, 2024.

## Results

### Incidence of iGAS

**Figure 1.**
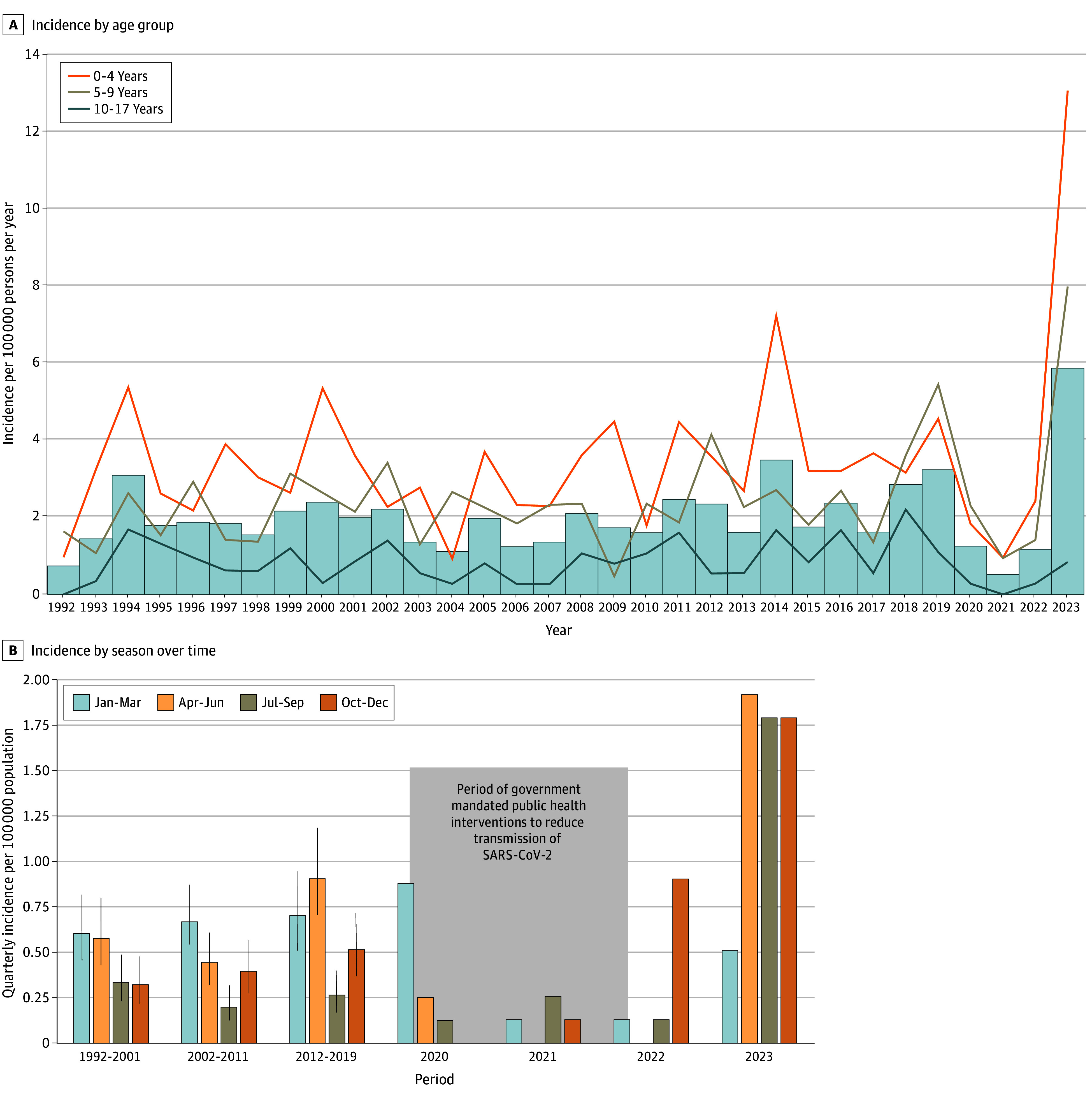
Incidence of Invasive Group A Streptococcal Infections in Children in Toronto and Peel Region, Canada A, Bars indicate overall incidence. B, Pandemic lockdown began on March 16, 2020, and the final pandemic interventions to reduce transmission (masking indoors and size of indoor gatherings) were lifted on March 21, 2022. Vertical lines indicate 95% CIs.

From January 1, 1992, to December 31, 2023, 498 iGAS cases were identified among children (300 [60.2%] male), including 241 children (48.4%) aged younger than 5 years, 166 children (33.3%) aged 5 to 9 years, and 91 children (18.3%) aged 10 to 17 years. Clinical data were available for all children, and *emm* types were available for 471 children (94.6%). The mean annual incidence from 1992 to 2023 was 3.43 events per 100 000 population per year in children aged younger than 5 years, 2.40 events per 100 000 population per year in children aged 5 to 9 years, and 0.81 events per 100 000 population per year in children aged 10 to 17 years (Figure 1A). The mean annual incidence was stable at 1.83 events per 100 000 population per year in 1992 to 2001 and 1.70 events per 100 000 population per year in 2002 to 2011 (IRR, 0.9 [95% CI, 0.7-1.2]), before increasing to 2.38 events per 100 000 population per year in 2012 to 2019 (IRR vs 1992-2011, 1.3 [95% CI, 1.1-1.6]). During the COVID-19 pandemic, the incidence declined to 0.51 events per 100 000 population in 2021 (compared to previous low in 1992-2011: IRR, 0.3 [95% CI, 0.1-0.8]) before increasing to 6.05 events per 100 000 population in 2023 (compared with previous high in 2012-2019: IRR, 2.5 [95% CI, 1.8-3.5]). Figure 1B illustrates the decline in iGAS incidence when public health measures were enacted to reduce SARS-CoV-2 transmission during the pandemic, as well as seasonal disease patterns over time.

### iGAS Disease Characteristics

Over the 32-year study period, the median (IQR) age of children with iGAS was 5.1 (2.7-8.6) years, and 151 children (30.7%) had underlying conditions (Table 1). The most common presentations were soft tissue infection (140 cases [28.1%]), bacteremia without focus (131 cases [26.3%]), bone and joint infection (97 cases [19.5%]), and pneumonia (66 cases [13.3%]).

**Table 1.** Characteristics Associated With iGAS Infections in Children by Age Group in Toronto and Peel Region, Canada, 1992-2023

Characteristic	Patients, No. (%)				*P* value
	Age 0-4 y (n = 241)	Age 5-9 y (n = 166)	Age 10-17 y (n = 91)	Total (N = 498)	
Sex					
Female	93 (38.6)	66 (39.8)	39 (42.9)	198 (39.8)	.73
Male	148 (61.4)	100 (60.2)	52 (57.1)	300 (60.2)	
Underlying conditions					
Any	58 (24.6)	60 (36.1)	33 (36.7)	151 (30.7)	.02^a^^,^^b^
Pulmonary	18 (7.6)	18 (10.8)	11 (12.2)	47 (9.5)	.24
Cardiac	8 (3.4)	9 (5.4)	0	17 (3.4)	.08
Dermatologic	12 (5.1)	8 (4.8)	3 (3.3)	23 (4.7)	.80
Neurologic	6 (2.5)	5 (3.0)	7 (7.8)	18 (3.7)	.07
Immunosuppression	9 (3.8)	11 (6.6)	2 (2.2)	22 (4.5)	.22
Kidney or liver	2 (0.9)	4 (2.4)	3 (3.3)	9 (1.8)	.19
Hematologic	4 (1.7)	3 (1.8)	0	7 (1.4)	.68
Lymphatic/vascular insufficiency	2 (0.9)	6 (3.6)	4 (4.4)	12 (2.4)	.06
Other	10 (4.2)	12 (7.2)	7 (7.8)	29 (5.9)	.33
VZV infection, prior 3 wk^c^	27 (11.2)	11 (6.6)	2 (2.2)	40 (8.0)	.02^b^
Trauma 1 wk prior to illness^d^	8 (3.8)	8 (5.4)	5 (6.0)	21 (4.8)	.68
Concurrent respiratory viral test findings^d^					
Positive	20 (20.4)	11(15.7)	5(13.9)	36 (17.6)	.15
Negative	24 (24.5)	13 (18.7)	3 (8.3)	40 (19.6)	
Not done	54 (55.1)	46 (65.8)	28 (77.8)	128 (62.7)	
Primary site of iGAS infection					
Soft tissue infection	66 (27.4)	41 (24.7)	33 (36.3)	140 (28.1)	.13
Bacteremia without focus	79 (32.8)	37 (22.3)	15 (16.5)	131 (26.3)	.004^a^^,^^b^
Bone or joint	35 (14.5)	45 (27.1)	17 (18.7)	97 (19.5)	.007^a^
Pneumonia	39 (16.2)	19 (11.4)	8 (8.8)	66 (13.3)	.17
Upper respiratory tract^e^	16 (6.6)	17 (10.2)	10 (11.0)	43 (8.6)	.31
Other^f^	6 (2.5)	7 (4.2)	8 (8.8)	21 (4.2)	.04^b^
Severe infections					
STSS	11 (4.6)	12 (7.2)	6 (6.6)	29 (5.8)	.51
NF	4 (1.7)	4 (2.4)	4 (4.4)	12 (2.4)	.35
Positive blood culture^g^	180 (74.7)	120 (72.3)	62 (68.1)	362 (72.7)	.48
Clindamycin resistance^f^	4 (3.0)	4 (3.8)	2 (3.4)	9 (3.0)	.69
*emm* type^h^					
*emm*1	83 (37.1)	68 (43.0)	31 (34.8)	182 (38.6)	.39
*emm*12	44 (19.6)	24 (15.2)	7 (7.9)	75 (15.9)	.04^b^^,^^i^
*emm*4	16 (7.1)	12 (7.6)	3 (3.4)	31 (6.6)	.34
*emm*3	12 (5.4)	10 (6.3)	7 (7.9)	29 (6.2)	.71
Other	69 (30.8)	44 (27.9)	41 (46.1)	154 (32.7)	.01^b^^,^^i^
Treatment					
Surgery^d^^,^^j^	53 (22.8)	60 (37.3)	35 (38.5)	148 (30.5)	.002^a^^,^^b^
IVIG^d^	21 (10.2)	18 (12.1)	7 (8.9)	46 (10.6)	.74
Outcome					
ICU admission	40 (17.1)	30 (18.5)	16 (17.6)	86 (17.7)	.95
30-d Mortality	2 (0.4)	4 (2.4)	4 (4.4)	10 (2.0)	.11

Abbreviations: ICU, intensive care unit; iGAS, invasive group A *Streptococcus*; IVIG, intravenous immunoglobulin; NF, necrotizing fasciitis; STSS, streptococcal toxic shock syndrome; VZV, varicella zoster virus infection.

^a^ Pairwise difference between ages 0 to 4 years and 5 to 9 years: *P* < .05.

^b^ Pairwise difference between ages 0 to 4 years and 10 to 17 years: *P* < .05.

^c^ Among patients with preceding chickenpox, 2 developed NF and 1 developed STSS.

^d^ Percentages vary because data are not available for all cases (variables with data for <97% of cases include: trauma [data available for 441 cases]; concurrent viral respiratory infection [204 cases since 2013]; clindamycin resistance [301 cases since 2002]; and IVIG therapy [434 cases]).

^e^ iGAS infections associated with the upper respiratory tract include pharyngitis, cervical adenitis, parapharyngeal and retropharyngeal abscesses, sinusitis, otitis, or periorbital cellulitis. These (and all other) infections were eligible only when a sterile culture yielded GAS and there was evidence of systemic disease (32 of 43 upper respiratory infections had a positive blood culture).

^f^ Other iGAS infection sites included meningitis, peritoneal infections, genitourinary infections, puerperal sepsis, and endocarditis.

^g^ Overall, blood cultures were obtained in 441 cases: 216 in children aged 0 to 4 years, 144 in children aged 5 to 9 years, and 81 in children aged 10 to 17 years.

^h^ Isolates and *emm* types were available for 471 cases (95%); individual *emm* types that comprised more than 5% of total cases are listed.

^i^ Pairwise difference between ages 5 to 9 years and 10 to 17 years: *P* < .05.

^j^ The most common surgical procedures were debridement (57 patients), athrotomy (25 patients), chest tube insertion/pleural window (17 patients), and incision and drainage (13 patients).

There were few differences in risk factors or clinical characteristics between age groups (Table 1). Children aged younger than 5 years were more likely to have chickenpox in the 3 weeks prior to iGAS than children aged 10 to 17 years (27 of 241 children [11.2%] vs 2 of 91 [2.2%]; OR, 5.61 [95% CI, 1.31-24.11]), and more likely to have bacteremia without focus than older children (79 of 241 children [32.8%] vs 52 of 257 children [20.2%]; OR, 1.92 [95% CI, 1.28-2.89]). Children aged 5 to 9 years were more likely to have bone or joint infections than those younger than 5 years (45 of 166 children [27.1%] vs 35 of 241 children [14.5%]; OR, 2.19 [95% CI, 1.33-3.60]). Surgery as therapy for iGAS occurred less commonly in children younger than 5 years than in older children (53 of 233 children [22.7%] vs 95 of 252 children [37.7%]; OR, 0.49 [95% CI, 0.33-0.72]).

### Comparison of Time Periods

**Figure 2.**
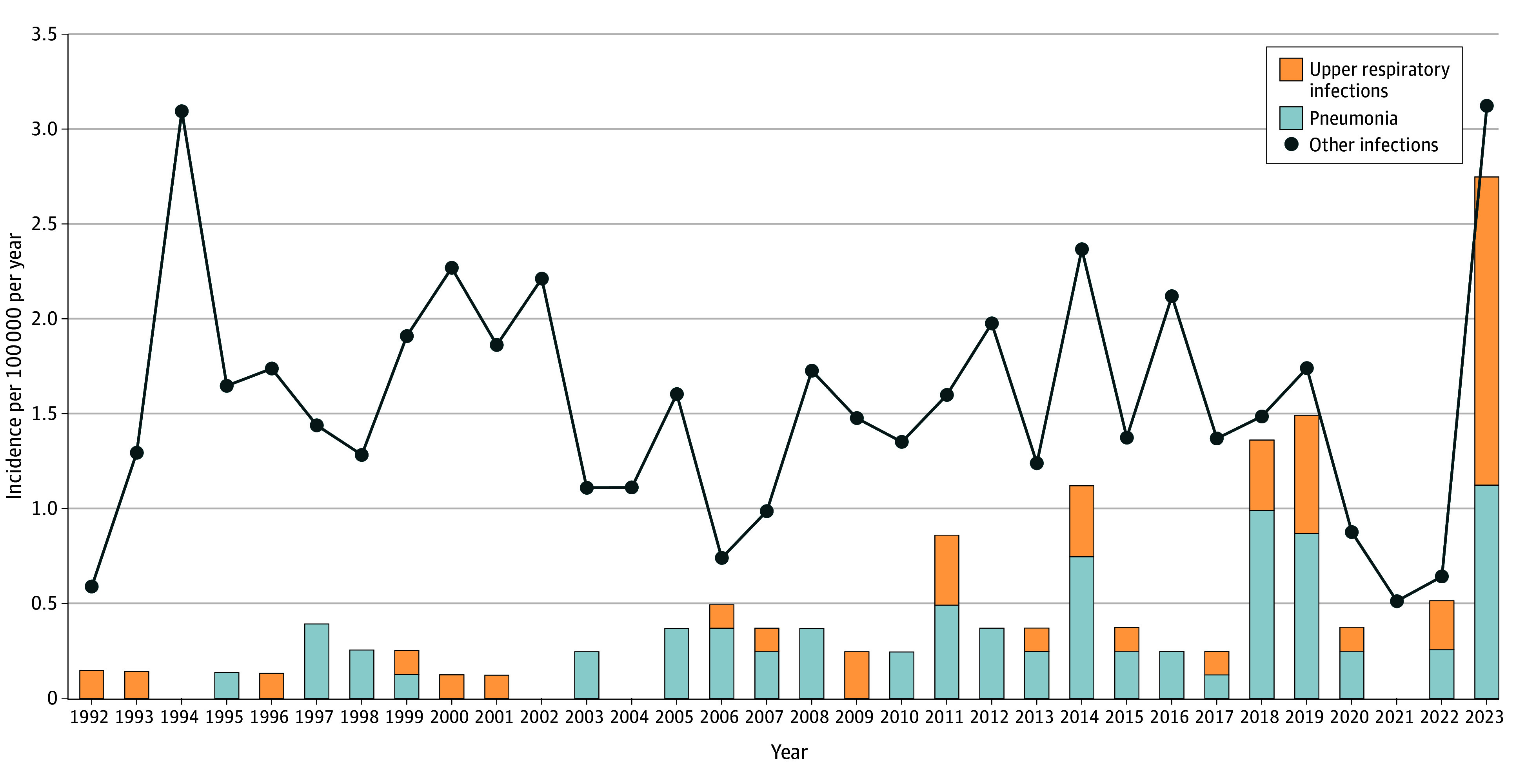
Distribution of Diagnoses in Pediatric Cases of Invasive Group A Streptococcal Disease in Toronto and Peel Region, Canada Upper respiratory tract infections include but are not limited to complicated pharyngitis, bacteremic periorbital cellulitis, and cervical adenitis.

There were no significant differences in the age distribution of iGAS infections over time (Table 2). Temporally associated with the implementation of publicly funded routine varicella zoster virus immunization in 2004,^20^ the proportion of iGAS complicating varicella zoster virus infection declined from 23 of 137 cases (16.8%) in 1992 to 2001 to 2 of 223 cases (0.9%) in 2012 to 2023 (*P* < .001). The incidence of iGAS with a primary respiratory tract focus increased over time (Figure 2). Pneumonia occurred in 60 of 323 cases (18.6%) from 2005 onwards, compared with 6 to 175 cases (3.4%) from 1992 to 2004 (OR, 6.43 [95% CI, 2.72-15.20]). iGAS infections of the upper respiratory tract (eg, complicated pharyngitis, periorbital cellulitis) were a significantly more common presentation during 2022 to 2023 than in prior decades (Table 2 and Figure 2).

**Table 2.** Characteristics Associated With iGAS Infections in Children by Time Period in Toronto and Peel Region, Canada

Characteristic	Patients, No. (%)					*P* value
	1992-2001 (n = 137)	2002-2011 (n = 138)	2012-2021 (n = 167)	2022-2023 (n = 56)	Total (N = 498)	
Sex						
Female	53 (38.7)	54 (39.1)	68 (40.7)	23 (41.1)	198 (39.8)	.97
Male	84 (61.3)	84 (60.9)	99 (59.3)	33 (58.9)	300 (60.2)	
Age group, y^a^						
0-4	73 (53.3)	62 (44.9)	74 (44.3)	32 (57.1)	241 (48.4)	.20
5-9	41 (29.9)	46 (33.3)	59 (35.3)	20 (35.7)	166 (33.3)	.81
10-17	23 (16.8)	30 (21.7)	34 (20.4)	4 (7.1)	91 (18.3)	.09
Underlying conditions						
Any	27 (20.2)	60 (44.1)	55 (33.1)	9 (16.1)	151 (30.7)	<.001^b^^,^^c^
Pulmonary	14 (10.5)	20 (14.7)	12 (7.2)	1 (1.8)	47 (9.6)	.03^b^^,^^d^
Cardiac	2 (1.5)	5 (3.7)	10 (6.0)	0	17 (3.5)	.08
Dermatologic	5 (3.7)	4 (2.9)	14 (8.4)	0	23 (4.7)	.03^c^
Neurologic	0	4 (2.9)	13 (7.8)	1 (1.8)	18 (3.7)	.003
Immunosuppression	3 (2.2)	9 (6.6)	7 (4.2)	3 (5.4)	22 (4.5)	.36
Kidney or liver dysfunction	1 (0.8)	4 (2.9)	3 (1.8)	1 (1.8)	9 (1.8)	.64
Hematologic	1 (0.8)	1 (0.7)	2 (1.2)	3 (5.4)	7 (1.4)	.10
Lymphatic or vascular	2 (1.5)	6 (4.4)	1 (0.6)	3 (5.4)	12 (2.4)	.07
Other	4 (3.0)	17 (12.5)	7 (4.2)	1 (1.8)	29 (5.9)	.002^b^
VZV infection in prior 3 wk	23 (16.8)	15 (10.9)	2 (1.2)	0	40 (8.0)	<.001^b^^,^^d^
Trauma 1 wk prior to illness^e^	10 (7.5)	0	4 (2.7)	7 (12.5)	21 (4.8)	.001^b^^,^^c^
Concurrent viral respiratory findings, No./total No. (%)^e^						
Positive	NA	NA	18/42 (42.9)	18/34 (52.9)	36/76 (47.4)	.39
Negative	NA	NA	24/42 (57.1)	16/34 (47.1)	40/76 (52.6)	
Primary site of iGAS infection						
Soft tissue infection	49 (35.8)	44 (31.9)	38 (22.8)	9 (16.1)	140 (28.1)	.01^b^^,^^d^
Bacteremia without focus	38 (27.7)	38 (27.5)	44 (26.4)	11 (19.6)	131 (26.3)	.68
Bone and joint	35 (25.6)	22 (15.9)	33 (19.8)	7 (12.5)	97 (19.5)	.12
Pneumonia	4 (2.9)	19 (13.8)	32 (19.2)	11 (19.6)	66 (13.3)	<.001^d^
Upper respiratory tract	6 (4.4)	7 (5.1)	15 (9.0)	15 (26.8)	43 (8.6)	<.001^b^^,^^c^^,^^d^
Other	5 (3.7)	8 (5.8)	5 (3.0)	3 (5.4)	21 (4.2)	.62
Severe infections						
STSS	0	10 (7.3)	14 (8.4)	5 (8.9)	29 (5.8)	.007^d^
NF	2 (1.5)	2 (1.5)	4 (2.4)	4 (7.1)	12 (2.4)	.19
Positive blood culture^f^	96 (70.1)	100 (72.2)	135 (80.8)	31 (55.4)	362 (72.7)	.006^b^^,^^c^^,^^d^
Clindamycin resistance^e^	NA	6 (4.7)	3 (2.5)	0	9 (3.0)	.23
*emm* types^g^						
*emm*1	46 (35.1)	43 (33.6)	66 (42.0)	27 (49.1)	182 (38.6)	.15
*emm*12	21 (16.0)	16 (12.5)	20 (12.7)	18 (32.7)	75 (15.9)	.003^b^^,^^c^^,^^d^
*emm*4	13 (9.9)	8 (6.3)	9 (5.7)	1 (1.8)	31 (6.8)	.13
*emm*3	8 (6.1)	11 (8.6)	10 (6.4)	0	29 (6.2)	.18
Other *emm* types	43 (32.8)	50 (39.1)	52 (33.1)	9 (16.4)	154 (32.7)	.03^b^^,^^c^^,^^d^
Treatment						
Surgery^e^	50 (39.1)	41 (30.4)	42 (25.3)	15 (26.8)	148 (30.5)	.08
IVIG^e^	9 (11.4)	17 (12.7)	10 (6.1)	10 (17.9)	46 (10.6)	.06
Outcomes						
ICU admission	12 (9.2)	27 (20.0)	32 (19.3)	15 (26.8)	86 (17.7)	.01^d^
30-d Mortality	3 (2.2)	2 (1.4)	2 (1.2)	3 (5.4)	10 (2.0)	.21

Abbreviations: ICU, intensive care unit; iGAS, invasive group A *Streptococcus*; IVIG, intravenous immunoglobulin; NA, not available; NF, necrotizing fasciitis; STSS, streptococcal toxic shock syndrome; VZV, varicella zoster virus infection.

^a^ Overall P = .22.

^b^ Pairwise difference between 2022 to 2023 and 2002 to 2011: *P* < .05.

^c^ Pairwise difference between 2022 to 2023 and 2012 to 2021: *P* < .05.

^d^ Pairwise difference between 2022 to 2023 and 1992 to 2001: *P* < .05.

^e^ Percentages vary because data are not available for all cases (variables with data for <97% of cases include: trauma [data available for 441], clindamycin resistance [data for 301 cases since 2002], and IVIG therapy,[data for 434 cases]).

^f^ Overall, blood cultures were obtained in 441 cases: 118 in 1992 to 2001, 120 in 2002 to 2011, 153 in 2012 to 2021, and 50 in 2022/23.

^g^ Individual *emm* types that comprised more than 5% of total cases are listed; for *emm* types listed, there were 471 cases with data.

### Association of Respiratory Viral Infections and iGAS

Results of testing of nasopharyngeal swabs and aspirates for respiratory viruses at iGAS presentation were not available prior to 2013. From 2013 to 2021, 42 of 148 children with iGAS (28.4%) had some testing for respiratory viruses at the time of iGAS presentation (eTable 2 in Supplement 1). Of 42 patients tested, 18 (42.9%) had viruses detected (including 2 coinfections). In 2022 to 2023, 34 of 56 children with iGAS (60.7%) had concurrent testing, and 18 (52.9%) had viruses detected (3 coinfections). Viruses identified included influenza A (9 children), respiratory syncytial virus (8 children), human metapneumovirus (7 children), entero/rhinovirus (7 children), SARS-CoV-2 (5 children), parainfluenza (3 children), and influenza B (2 children). Children younger than 5 years whose nasopharyngeal swabs or aspirates yielded a respiratory virus were more likely to have pneumonia than those whose test results were negative (11 of 20 children [55.0%] vs 1 of 24 children [4.2%]; OR, 28.11 [95% CI, 3.15-250.52]); this difference was not apparent in older children (5 of 16 children [31.3%] vs 4 of 16 children [25.0%]; OR, 1.35 [95% CI, 0.27-7.03]).

### Microbiology

GAS was most commonly identified in blood cultures (362 of 498 cultures [72.7%]). The proportion of iGAS cases with positive blood cultures increased from 196 of 275 cultures (71.2% ) in 1992 to 2011 to 135 of 167 cultures (80.8%) in 2012 to 2021 (*P* = .03) but was lower (31 of 56 cultures [55.4%]) in 2022 to 2023 (*P* = .02 and *P* < .001 compared to 1992-2011 and 2012-2021, respectively) (Table 2). In 136 children (27.3%) with blood cultures that were not done or had no bacterial growth, the most common positive sterile site cultures were tissue or aspirates (60 children [44.1%]), joint fluid (45 children [33.1%]), and pleural or peritoneal fluid (30 children [22.1%]). Clindamycin resistance remained uncommon in all age groups and did not change over time (Table 2).

**Figure 3.**
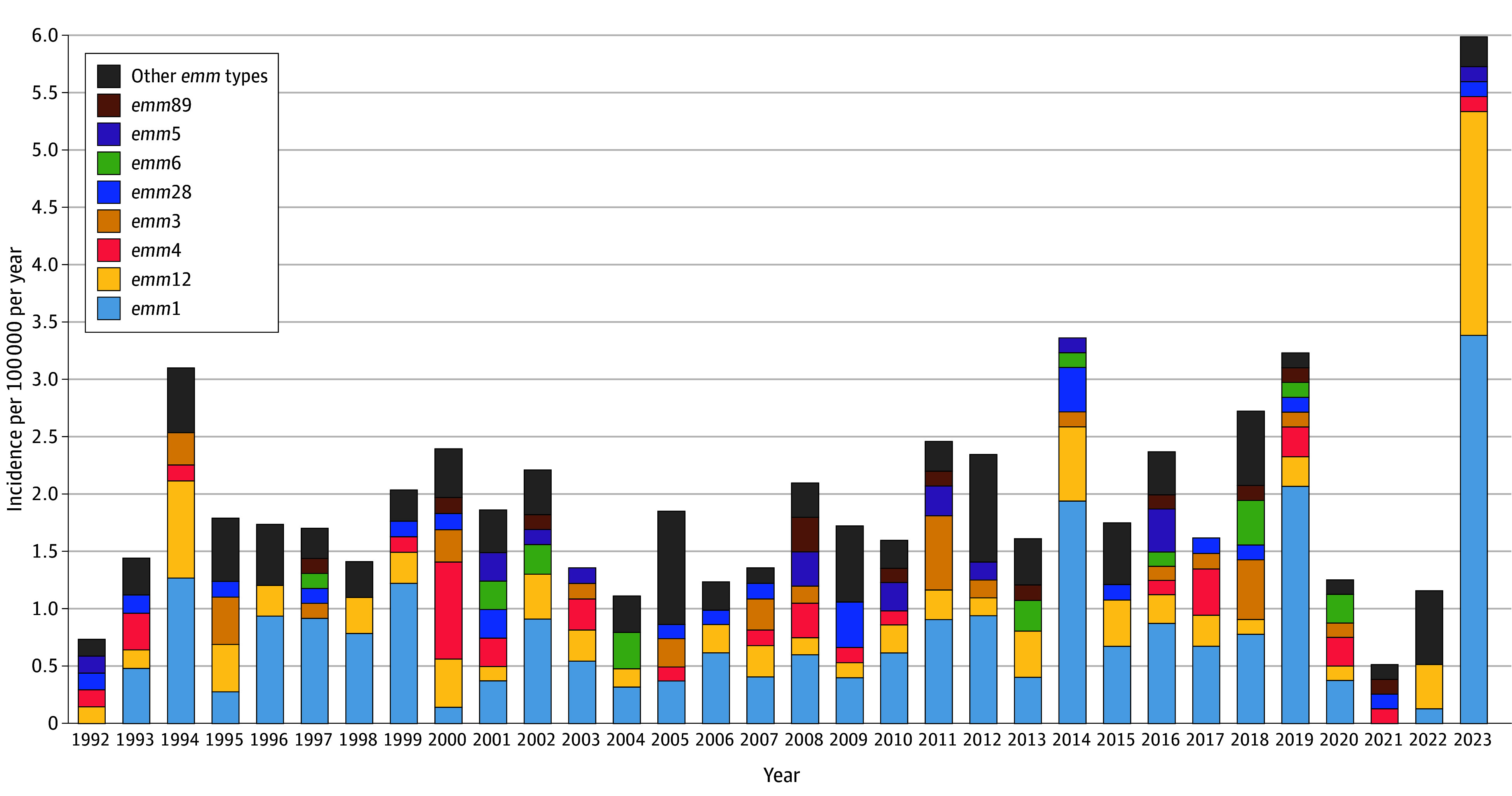
Annual Incidence of Invasive Group A *Streptococcus* Cases in Children in Toronto and Peel Region, Canada, by *emm* Type The most common 8 *emm* types overall are shown individually.

The most common *emm* types were *emm*1 (182 of 471 isolates [38.6%]) and *emm*12 (75 isolates [15.9%]) (Table 2; eTable 1 in Supplement 1). The incidence of disease due to *emm*1 was 0.64 events per 100 000 population per year in 1992 to 2001 and 0.57 events per 100 000 population per year in 2002 to 2011 (IRR, 0.9 [95% CI, 0.6-1.4]), then increased to 1.0 events per 100 000 population per year in 2012 to 2019 (IRR, 1.7 [95% CI, 1.2-2.4]). During the pandemic, the incidence declined sharply, with a single case identified between February 2020 and November 2022 (Figure 3). M1_UK_ was not identified prior to 2019, but comprised 32 of 46 *emm*1 isolates (70.0%) from 2019 to 2023. *emm*12 was present but uncommon from 1992 to 2022, with a median (range) of 2 (0-6) cases annually and infecting somewhat younger children (median [IQR] age, 4.3 [1.7-6.7] years vs 5.6 [3.0-9.1] years for other *emm* types; *P* = .01). However, during 2023, 15 of 46 iGAS cases (32.6%) were due to *emm*12. In contrast, *emm*4 and *emm*3 (the third and fourth most common *emm* types) were each associated with a median (range) of 1 (0-6) and 1 (0-5) cases annually, respectively, before the pandemic but did not increase in 2022 to 2023 (eTable 1 in Supplement 1).

Compared with other *emm* types, *emm*1 was more likely to be associated with a diagnosis of pneumonia (33 of 182 cases [18.1%] vs 29 of 289 cases [10.0%]; OR, 1.99 [95% CI, 1.16-3.40]) and bone or joint infections (46 cases [25.3%] vs 48 cases [16.6%]; OR, 1.70 [95% CI, 1.08-2.68]) but less commonly with bacteremia without focus (30 cases [16.5%] vs 90 cases [31.1%]; OR, 0.43 [95% CI, 0.27-0.68]); *emm*4 was associated with bacteremia without focus (20 of 31 [64.5%] vs 101 of 440 cases [23.0%]; OR, 6.10 [95% CI, 2.83-13.16]). Overall, 437 of 471 isolates (92.8%) were of *emm* types included in the 30-valent M protein-based vaccine, with no significant change over time (eTable 1 in Supplement 1).^21,22^

### Illness Severity and Outcomes

STSS occurred in 29 of 498 children (5.8%), NF in 12 children (2.4%), and intensive care unit (ICU) admissions in 86 children (17.7%); the 30-day case fatality rate was 2.0% (10 of 498 patients) (Table 1 and Table 2). Children with pneumonia, compared with children with other sites of infection, were more likely to have STSS (12 of 66 children [18.2%] vs 17 of 432 children [3.9%]; OR, 5.43 [95% CI, 2.46-11.97]) and to require ICU admission (33 children [50.0%] vs 53 children [12.6%]; OR, 6.94 [95% CI, 3.96-12.18]) (eTable 3 in Supplement 1).

There were no significant differences in the proportions of severe outcomes between age groups. However, fewer ICU admissions and cases of STSS were observed in 1992 to 2001 compared with subsequent time periods (Table 2). *emm*1 was associated with a higher risk of ICU admission than other *emm* types (41 of 179 children [22.9%] vs 43 of 284 children [15.1%]; OR, 1.67 [95% CI, 1.03-2.68]). We were unable to detect a difference in a diagnosis of pneumonia, STSS, or NF, or an increased risk of ICU admission or death in infections due to M1_UK_ compared with other *emm*1 strains.

## Discussion

This case series examines the epidemiology of iGAS in children in south-central Ontario over the past 3 decades, highlighting changes in incidence and strain distribution, and the impact of the public health measures associated with the COVID-19 pandemic. Most characteristics of iGAS did not change. However, the incidence of iGAS with a primary respiratory site of infection increased, while that of primary soft tissue and other nonrespiratory iGAS infections did not decrease despite a significant reduction in cases occurring as a complication of chickenpox. The primary age-related differences observed included a higher rate of bacteremia without focus, *emm*12 infections, iGAS complicating chickenpox in younger vs older children, and higher rates of bone or joint infections in children aged 5 to 9 years. Distribution of *emm* types varied over time and *emm*1 was notably associated with a higher incidence of pneumonia and ICU admission. The proportion of iGAS cases with STSS and requiring ICU admission increased after 2001; case fatality rates were too low to assess whether change occurred.

The resurgence in incidence after the pandemic occurred later in our population compared with the United Kingdom or the Netherlands.^23,24^ However, it is also likely a result of an increased pool of nonimmune children, resulting from public health measures enacted to reduce SARS-CoV-2 transmission also reducing GAS transmission, particularly of *emm*1 strains, during the pandemic.^11,25^ The postpandemic resurgence of viral respiratory infections, which are known to predispose to iGAS, also likely contributed to increased disease incidence.^7,26,27^

Reports of postpandemic increases in severe iGAS associated with meningitis in the Netherlands and ICU admission in Belgium identified the M1_UK_ variant as the predominant cause of disease.^8,9^ However, in studies of postpandemic increases including less severe iGAS cases, the contributions of different *emm* types have differed: from predominantly *emm*1 (the Netherlands), to contributions from *emm*1 and *emm*12 (our study, the United Kingdom, the Netherlands, Minnesota, and Colorado), to predominantly *emm*12 (Houston, Texas), to increases more broadly across all *emm* types (New Zealand, Australia).^7,10,23,24,28,29,30^ The increased prevalence of *emm*12 in asymptomatic throat carriage in the Netherlands might suggest that *emm*12 contributed to our increasing rates of iGAS infections of the upper respiratory tract; however, in our data, *emm*12 was not associated with respiratory tract sites of infections.^30^

Reports of increasing clindamycin resistance have raised concerns about the continuing benefit of adjunctive clindamycin therapy for STSS.^31,32^ The substantial global variability in the prevalence of clindamycin nonsusceptibility is not completely understood; however, one important factor is variability in the prevalence of different *emm* types, in which macrolide and lincosamide resistance varies widely.^33^ In a 2021 publication from the US ABC surveillance system summarizing data from 2006 to 2017,^33^ clindamycin nonsusceptibility in children younger than 5 years was not substantially different than in our young children (5.7% vs 3.0%) and significantly lower than that in adults, likely a result of differences in *emm* type distributions in adult vs pediatric iGAS infections.^24,33^

The link between *emm*1 and severe iGAS or scarlet fever in the late 1980s^2^ was thought to be due to clonal changes and virulence factors, such as SpeA, DNAase, and streptolysin O.^34^ Our data confirm that *emm*1 infections continue to be associated with more severe disease. The M1_UK_ sublineage has also been found to have upregulation of SpeA compared with other *emm*1 type sublineages; however, other lineages of *emm*1 also express SpeA.^35^ Limited power is likely the reason we did not identify increased severity associated with M1_UK_, but diversity in and multiplicity of virulence factors in different GAS clades might also contribute.

In our population, there was an increased incidence of iGAS pneumonia that began in the early 2000s, while the incidence of iGAS associated with other sites of infection did not change. As in other jurisdictions, the increase in GAS pneumonia occurred in temporal association with the introduction of pneumococcal conjugate vaccines and may have been a result of the associated changes in respiratory flora.^36,37^ Another possibility might be that iGAS incidence overall was increasing, but the iGAS decline due to the introduction of the varicella vaccine counterbalanced this increase in soft tissue but not in respiratory infections. Compatible with this hypothesis, the introduction of a varicella vaccine program in Israel was associated with a decline in overall iGAS incidence.^38^ Recent studies in Belgium^8^ and Colorado^26^ have reported a significant increase in lower respiratory tract infections due to GAS during the postpandemic surge, similar to that in our population.

Although some non–M-protein vaccines against GAS are in development, current clinical-phase vaccine candidates focus on the GAS M protein, with antigens from the hypervariable N-terminal or conserved C-terminal epitopes. The most advanced is a 30-valent M protein–based vaccine, which demonstrated good tolerance, no autoimmune response or cross-reactivity, high immunogenicity, and selective GAS killing in a phase 1 trial.^21,22^ This vaccine covers more than 90% of strains that caused pediatric iGAS in our population area. However, in populations in low- and middle-income countries, where strain distribution is different and diversity greater, other vaccines may be needed to provide adequate strain coverage.^39,40^

Except for the correlation between *emm*1 and ICU admission, we did not identify associations between specific *emm* types and severity of disease. Importantly, the genomic evolution of *emm* types and the virulence factors they acquire over time can contribute to severe disease manifestations, which are thus not necessarily determined by the specific *emm* type alone.^34^

### Limitations

This study has limitations. It reports on disease in 1 geographic area in a high-income country, limiting its generalizability. Our data were collected by medical record review and some are incomplete. The variation in the proportion of cases with positive blood cultures over time may represent variation in the application of definitions of nonblood sterile site by different laboratories and staff; however, this would result in minimizing the estimate of the increase in incidence prior to the pandemic. Viral respiratory testing methods were different in different hospitals and laboratories and changed over time, limiting interpretation of associations between specific viruses and iGAS. Our sample size also limited our power to detect associations between *emm* type and severity of illness. We also did not assess other virulence markers that might contribute to the severity of iGAS. In addition, we were not able to assess the impact of iGAS disease among with children who were dead on arrival at health care facilities: these cases are legal cases in which data cannot be shared with research studies. Between 1992 and 2005, positive iGAS cultures from autopsies of some of these cases (10 cases) were reported to us; some, but not all, of these cases likely had iGAS as a cause of death.^41^

## Conclusions

In this population-based case series of pediatric iGAS infections in Toronto and Peel Region of Canada over 3 decades, iGAS incidence increased significantly in the decade prior to the COVID-19 pandemic. It remains unclear whether the postpandemic resurgence represents a continuation of this increase, a temporary effect of postpandemic conditions, or a combination of both. Despite this increase, disease characteristics remained largely unchanged, except for a notable increase in iGAS infections of the upper and lower respiratory tract. Assessments of burden of disease in preparation for the introduction of GAS vaccines should include consideration of cases in which children are dead on arrival to health care. This study also emphasizes the importance of considering age-specific factors in the clinical presentation of iGAS infections, reinforces the value of varicella vaccination in preventing iGAS, and highlights the potential for both respiratory viral and GAS vaccines to alleviate the burden of iGAS infections.

## Notes

Supplement 1. **eTable 1.** Distribution of *emm* Types of Isolates Associated With Pediatric Invasive GAS Infections, Toronto and Peel Region, 1992-2023 **eTable 2.** Respiratory Viruses Identified From Nasopharyngeal Swabs/Aspirates Obtained in Children Presenting With iGAS Infections, Toronto and Peel Region, Canada, 2013-2023 **eTable 3.** Association of Presentations and Emm Types With Outcomes in Pediatric iGAS Infections, Toronto and Peel Region, Canada, 1992-2023

Supplement 2.
Nonauthor Members of the Toronto Invasive Bacterial Diseases Network

Supplement 3.
Data Sharing Statement
