# Supplementary material for: Invasive Group A Streptococcal Infection in Children, 1992-2023
Source: JAMA Netw Open. 2025 Apr 1;8(4):e252861. doi: 10.1001/jamanetworkopen.2025.2861 (PMC11962665; doi:10.1001/jamanetworkopen.2025.2861)
Supplement: Supplement 1. — eTable 1. Distribution of emm Types of Isolates Associated With Pediatric Invasive GAS Infections, Toronto and Peel Region, 1992-2023 eTable 2. Respiratory Viruses Identified From Nasopharyngeal Swabs/Aspirates Obtained in Children Presenting With iGAS Infections, Toronto and Peel Region, Canada, 2013-2023 eTable 3. Association of Presentations and Emm Types With Outcomes in Pediatric iGAS Infections, Toronto and Peel Region, Canada, 1992-2023 [file jamanetwopen-e252861-s001.pdf]

Supplemental Online Content

Dabaja-Younis H, Kandel C, Green K, et al; for the Toronto Invasive Bacterial Diseases Network. Invasive group A streptococcal infection in children, 1992-2023. *JAMA Netw Open*. 2025;8(4):e252861. doi:10.1001/jamanetworkopen.2025.2861

eTable 1. Distribution of *emm* Types of Isolates Associated With Pediatric Invasive GAS Infections, Toronto and Peel Region, 1992-2023

eTable 2. Respiratory Viruses Identified From Nasopharyngeal Swabs/Aspirates Obtained in Children Presenting With iGAS Infections, Toronto and Peel Region, Canada, 2013-2023

eTable 3. Association of Presentations and Emm Types With Outcomes in Pediatric iGAS Infections, Toronto and Peel Region, Canada, 1992-2023

This supplemental material has been provided by the authors to give readers additional information about their work.

© 2025 Dabaja-Younis H et al. *JAMA Network Open*.

eTable 1: Distribution of *emm* types of isolates associated with pediatric invasive GAS infections, Toronto and Peel region, 1992-2023

emm type	Total, 1992-2023 N=471	1992-2001 N=131	2002-2011 N=128	2012-2021 N=157	2022-2023 N=55
Included in 30-valent vaccine	437 (92.8)	121 (92.4)	117 (91.4)	146 (93.0)	53 (96.4)
1	182 (38.6)	46 (35.1)	43 (33.6)	66 (42.0)	27 (49.0)
12	75 (15.9)	21 (16.0)	16 (12.5)	20 (12.7)	18 (32.7)
4	31 (6.6)	13 (9.9)	8 (6.3)	9 (5.7)	1 (1.8)
3	29 (6.2)	8 (6.1)	11 (8.6)	10 (6.4)	0
28	23 (4.9)	8 (6.1)	6 (4.7)	8 (5.1)	1 (1.8)
5	17 (3.6)	3 (2.3)	8 (6.3)	5 (3.2)	1 (1.8)
6	17 (3.6)	3 (2.3)	4 (3.1)	10 (6.4)	0
89	12 (2.5)	2 (1.5)	5 (3.9)	5 (3.2)	0
49	9 (1.9)	3 (2.3)	0	2 (1.3)	4 (8.0)
87	7 (1.5)	2 (1.5)	0	4 (2.5)	1 (1.8)
11	6 (1.3)	3 (2.3)	2 (1.6)	1 (0.64)	0
2	5 (1.1)	1 (0.76)	2 (1.6)	2 (1.3)	0
75	5 (1.1)	1 (0.76)	4 (3.1)	0	0
118	5 (1.1)	2 (1.5)	3 (2.3)	0	0
9	4 (0.85)	0	1 (0.77)	3 (1.9)	0
73	4 (0.85)	0	2 (1.5)	2 (1.3)	0
61	3 (0.64)	1 (0.76)	2 (1.5)	0	0
81	3 (0.64)	1 (0.76)	0	2 (1.3)	0
183	3 (0.64)	2 (1.5)	0	1 (0.64)	0
56	2 (0.42)	1 (0.76)	0	1 (0.64)	0
58	2 (0.42)	1 (0.76)	0	0	1 (1.8)
63	2 (0.42)	2 (1.5)	0	0	0
208	2 (0.42)	1 (0.76)	0	1 (0.64)	0
15	1 (0.21)	0	1 (0.77)	0	0
18	1 (0.21)	0	1 (0.77)	0	0
22	1 (0.21)	1 (0.76)	0	0	0
33	1 (0.21)	0	0	1 (0.64)	0
44	1 (0.21)	0	0	1 (0.64)	0
48	1 (0.21)	0	0	1 (0.64)	0
50	1 (0.21)	0	0	1 (0.56)	0
65	1 (0.21)	1 (0.76)	0	0	0
67	1 (0.21)	0	0	1 (0.64)	0
78	1 (0.21)	1 (0.76)	0	0	0
83	1 (0.21)	0	0	1 (0.64)	0
88	1 (0.21)	0	1 (0.77)	0	0
92	1 (0.21)	1 (0.76)	0	0	0
106	1 (0.21)	0	0	1 (0.64)	0
112	1 (0.21)	0	1 (0.77)	0	0
119	1 (0.21)	1 (0.76)	0	0	0
123	1 (0.21)	0	1 (0.77)	0	0
151	1 (0.21)	0	0	0	1 (1.8)
165	1 (0.21)	0	0	1 (0.64)	0
168	1 (0.21)	0	1 (0.77)	0	0
170	1 (0.21)	0	1 (0.77)	0	0
177	1 (0.21)	1 (0.76)	0	0	0
232	1 (0.21)	0	0	1 (064)	0

© 2025 Dabaja-Younis H et al. *JAMA Network Open*.

eTable 2: Respiratory viruses identified from nasopharyngeal swabs/aspirates obtained in children presenting with iGAS infections, Toronto and Peel region, Canada, 2013-2023

Respiratory virus identified	2013-2021 (18/42 children tested had a virus detected)[a]	2022-2023 (18/34 children tested had a virus detected)[b]
Single virus identified		
Influenza	6 influenza A, 1 influenza B	2 influenza A, 1 influenza B
Respiratory syncytial virus (RSV)	4	3
Human metapneumovirus (HMPV)	2	3
Entero/Rhinovirus	2	1
SARS-CoV-2	1	3
Parainfluenza	0	2
Two viruses identified	2 HMPV + Entero/rhinovirus	1 influenza A + Entero/rhinovirus<br>1 Parainfluenza + Entero/rhinovirus<br>1 RSV + SARS-CoV-2

[a]Viral testing was performed in 8 laboratories from 2013-2021 and included: 21 specimens tested by a multiplex polymerase chain reaction (PCR) panel authorized by Health Canada and manufactured by Seegene®, Luminex®, or BD® (BDMAXFull); 4 tested by a validated laboratory developed multiplex panel detecting 13 respiratory viruses; 2 tested by a combined influenza and RSV PCR test authorized by Health Canada; 4 tested for influenza by an authorized influenza PCR test and for RSV by an authorized RSV enzyme immunoassay kit; 9 tested by direct fluorescent antigen (Simulfluor (influenza A/B, RSV, parainfluenza 1-3, adenovirus) monoclonal antibodies (Millipore) and hMPV monoclonal antibodies (Diagnostic Hybrids)); 2 tested by viral culture (R-Mix shell vials, Diagnostic Hybrids). Testing for SARS-CoV-2 was performed for separately from March 2020 onwards by a variety of Health Canada authorized PCR platforms.
[b]Viral testing performed by a multiplex polymerase chain reaction (PCR) respiratory virus panel authorized by Health Canada (manufactured by Seegene® or BD® (N=26 specimens), or a laboratory developed, validated multiplex PCR panel (N=4), or by a Health Canada authorized PCR test for influenza, RSV and SARS-CoV-2 (N=4).

© 2025 Dabaja-Younis H et al. *JAMA Network Open*.

eTable 3: Association of presentations and *emm* types with outcomes in pediatric iGAS infections, Toronto and Peel region, Canada, 1992-2023

	Infection severity/outcome							
	NF (Total=12)		STSS (Total=29)		ICU admission (Total=86)		Death (Total=11)	
	N (%)	OR (95% CI)	N (%)	OR (95%CI)	N (%)	OR (95%CI)	N (%)	OR (95%CI)
Main presentation								
Soft tissue infection (N=140)	-	-	8 (5.7)	1 (0.4-2.3)	19 (14)	0.7 (0.4-1.2)	1 (0.7)	0.3 (0.03-2.0)
Bacteremia without focus (N=131)	-	-	3 (2.2)	0.3 (0.1-1)	18 (14)	0.7 (0.4-1.3)	4 (3.1)	1.6 (0.5-5.6)
Upper respiratory tract (N=43)	-	-	2 (4.7)	0.8 (0.2-3.4)	6 (14)	0.8 (0.3-1.9)	0	NE
Bone/joint infection (N=97)	-	-	0	0.9 (0. 9-1)	4 (4.1)	0.2 (0.1-0.5)	0	NE
Pneumonia (N=67)	-	-	12 (18)	5.3 (2.4-12)	33 (49)	6.7 (3.9-12)	4 (6.0)	3.9 (1.1-14)
Other (N=21)	-	-	4 (9.5)	4.3 (1.3-14)	6 (29)	2.2 (0.8-6.1)	2 (1.0)	5.5 (1.1-27)
emm type (N=471)								
*emm*1 (N=182)	5 (2.7)	1.1 (0.4-3.6)	15 (8.2)	2.1 (0.9-4.5)	41 (23)	1.7 (1.0-2.7)	6 (3.3)	1.9 (0.6-6.4)
*emm*12 (N=75)	2 (2.7)	1 (0.2-4.8)	4 (5.3)	0.9 (0.3-2.7)	14 (18)	1 (0.6-2)	2 (2.7)	1.2 (0.2-5.5)
*emm*4 (N=31)	2 (6.5)	2.8 (0.6-13.6)	0	NE	5 (16)	0.9 (0.3-2.3)	2 (6.5)	3.2 (0.7-15)
*emm*3 (N=29)	2 (6.9)	3.2 (0.7-15.3)	1 (3.2)	0.6 (0.1-4.4)	6 (21)	1.2 (0.5-3)	0	NE
Other *emm* types (N=154)	1 (0.6)	0.2 (0.02-1.4)	7 (4.5)	0.7 (0.3-1.7)	18 (12)	0.5 (0.3-0.9)	1 (0.6)	0.2 (0.03-1.6)

Abbreviations: CI=confidence interval; ICU=intensive care unit; NE=not estimable; NF=necrotizing fasciitis; OR=odds ration; STSS=streptococcal toxic shock syndrome.

© 2025 Dabaja-Younis H et al. *JAMA Network Open*.
