# Supplementary material for: Invasive Group A Streptococcal Infection in Children, 1992-2023
Source: JAMA Netw Open. 2025 Apr 1;8(4):e252861. doi: 10.1001/jamanetworkopen.2025.2861 (PMC11962665; doi:10.1001/jamanetworkopen.2025.2861)
Supplement: Supplement 2. — Nonauthor Members of the Toronto Invasive Bacterial Diseases Network [file jamanetwopen-e252861-s002.pdf]

Supplemental Online Content: Nonauthor Collaborators
*First name, last name, and suffix (if applicable) are required and will appear in PubMed.

*Group Name(s): Toronto Invasive Bacterial Diseases Network							
***First Name and Middle Initial(s)**	***Last Name**	***Suffix (eg, Jr, III)**	Academic Degrees	Institution	Location (city, state/province, country)	Role or Contribution, eg, chair, principal investigator	Group (if more than 1 Group listed in the byline) and/or Subgroup (eg, Steering Committee)
Abdelbaset	Belhaj		MBBS	Scarborough Health Network	Toronto, Ontario, Canada	Site investigator	
Reena	Lovinsky		MD	Scarborough Health Network	Toronto, Ontario, Canada	Site investigator	
David	Rose		MD	Scarborough Health Network	Toronto, Ontario, Canada	Site investigator	
Danny	Chen		MD	Mackenzie Health	Richmond Hill, Ontario Can	Site investigator	
Ramzi	Fattouh		PhD	Unity Health Toronto	Toronto, Ontario, Canada	Co-investigator	
Sigmund	Krajden		MD	Unity Health Toronto	Toronto, Ontario, Canada	Site investigator	
Larissa	Matukas		MD	Unity Health Toronto	Toronto, Ontario, Canada	Site investigator	
Lee	Goneau		PhD	Dynacare	Brampton, Ontario, Canada	Site investigator	
Alainna	Jamal		MD	University of Toronto	Toronto, Ontario, Canada	Co-investigator	
Amna	Faheem		MBBS	Unity Health Toronto	Toronto, Ontario, Canada	Co-investigator	
Kevin	Katz		MD	North York General Hospital	Toronto, Ontario, Canada	Site investigator	
Mary Ann	MacDonald		MLT	Soldier's Memorial Hospital	Orillia, Ontario Canada	Site investigator	
Tony	Mazzulli		MD	Sinai Health	Toronto, Ontario, Canada	Site investigator	
Susan	Poutanen		MD	Sinai Health	Toronto, Ontario, Canada	Co-investigator	
Andrew	Simor		MD	Sunnybrook Health Sciences Centre	Toronto, Ontario, Canada	Site investigator	
Samira	Mubareka		MD	Sunnybrook Health Sciences Centre	Toronto, Ontario, Canada	Site investigator	
Samir	Patel		PhD	Public Health Ontario Laboratory	Toronto, Ontario, Canada	Site investigator	
Valerie	Sales		MD	Oak Valley Health	Markham, Ontario, Canada	Site investigator	
Sharon	Walmsley		MD	University Health Network	Toronto, Ontario, Canada	Site investigator	
Christie	Vermeiren		PhD	Shared Hospital Laboratory	Toronto, Ontario, Canada	Site investigator	
Deborah	Yamamura		MD	Hamilton Regional Laboratory Medici	Hamilton, Ontario, Canada	Site investigator	
