# Supplementary material for: Invasive Group A Streptococcal Infection in Children, 1992-2023
Source: JAMA Netw Open. 2025 Apr 1;8(4):e252861. doi: 10.1001/jamanetworkopen.2025.2861 (PMC11962665; doi:10.1001/jamanetworkopen.2025.2861)
Supplement: Supplement 3. — Data Sharing Statement [file jamanetwopen-e252861-s003.pdf]

Data Sharing Statement

Dabaja-Younis. Invasive Group A Streptococcal Infection in Children, 1992-2023. *JAMA Netw Open*. Published April 01, 2025. doi:10.1001/jamanetworkopen.2025.2861

Data
Data available: Yes
Data types: Deidentified participant data, Data dictionary
How to access data: Upon REB approval of submitted protocol. Access via allison.mcgeer@sinaihealth.ca
When available: With publication

Supporting Documents
Document types: None

Additional Information
Who can access the data: researchers whose proposed use of the data has been approved by a qualified Research ethics board and by the REB of Sinai Health System
Types of analyses: For specified purposes
Mechanisms of data availability: After approval of a proposal, and with a signed data transfer agreement
Any additional restrictions: None
